# Changes of Serum Melatonin, Interleukin-6, Homocysteine, and Complement C3 and C4 Levels in Patients With Depression

**DOI:** 10.3389/fpsyg.2020.01271

**Published:** 2020-06-23

**Authors:** Huai Tao, Xia Chen, Hongfei Zhou, Jinhua Fu, Qi Yu, Yong Liu

**Affiliations:** ^1^School of Medicine, Hunan University of Chinese Medicine, Changsha, China; ^2^Department of Orthopedics, The Second Xiangya Hospital of Central South University, Changsha, China; ^3^Department of Psychiatry, Hunan Provincial Brain Hospital, Changsha, China; ^4^National Clinical Research Center for Mental Disorders, Department of Psychaitry, The Second Xiangya Hospital of Central South University, China National Technology Institute on Mental Disorders, Hunan Key Laboratory of Psychiatry and Mental Health, Changsha, China

**Keywords:** depression, melatonin, interleukin-6, homocysteine, complement

## Abstract

**Objectives:**

Cytokine activation and low complement levels are common in depression patients. This study is aimed at investigating the clinical significance of changes in serum concentrations of melatonin (MT), interleukin-6 (IL-6), homocysteine (hcy), and complement C3 and C4 in depression patients and relationships of them with depression activity.

**Methods:**

A total of 95 depression patients, including first-episode group (*n* = 43) and recurrent group (*n* = 52), and 45 age- and gender-matched healthy controls (HC) were recruited. Serum levels of MT, IL-6, hcy, C3, and C4 in all samples were measured by using enzyme-linked immunosorbent assay (ELISA), chemiluminescence method, enzyme circulation method, and immuno-scatter turbidimetric assay, respectively.

**Results:**

The serum MT, IL-6, and hcy levels in the first-episode group (113.08 ± 5.06 pg/ml, 2.06 ± 0.12 ng/L, and 13.87 ± 0.45 μmol/L), and recurrent group (117.63 ± 4.63 pg/ml, 2.20 ± 0.12 ng/L, and 13.61 ± 0.46 μmol/L) were significantly higher than those in the control group (89.50 ± 5.10 pg/ml, 1.57 ± 0.06 ng/L, and 11.34 ± 0.40 μmol/L). The serum levels of C3 in the first-episode group (0.95 ± 0.02 ng/L) were significantly lower than those in the recurrent group (1.05 ± 0.03 ng/L) and control group (1.12 ± 0.03 ng/L). There was no significant difference in serum C4 level between each group.

**Conclusion:**

These results suggest that higher serum MT, IL-6, and hcy levels were correlated with pathogenesis of depression.

## Introduction

Depression, also known as depressive disorder, is a kind of mental and psychological disease characterized by listless, depression, and inferiority. Some depression patients may have self-harm or suicidal behavior, and the incidence rate of depression is rising toward younger age (World Health Organization, 2017). The pathological mechanisms of depression involve complicated physiological and psychological factors. Recent researches have reported that monoamine hormones, inflammatory cytokines, neurotrophic factors, and autoimmune markers are most likely to exist as biomarkers of depression (Miller and Raison, 2016), but further research is still needed. Melatonin (MT) is an amine hormone that is secreted from human pineal gland and released into blood. It can modulate the phase of circadian rhythms by binding to MT receptor in hypothalamic suprachiasmatic nucleus. The altered secretion mode of MT causes the disruption of circadian rhythms and then facilitates mood disorder in depression patients (Valdés-Tovar et al., 2018). Furthermore, MT can suppress the release of glutamate and decrease the inhibitory activity of glutamate on brain-derived neurotrophic factor to lower damage in the neurons of hippocampus, improving cognitive function of major depressive disorder (MDD) patients (Treadway et al., 2015). Moreover, a combination of anti-depressant drug buspirone with MT could improve the cognitive function of the major depression disorder patient when compared with the buspirone-alone group and the placebo group (Targum et al., 2015), suggesting the importance of MT in pathogenesis of depression.

However, interestingly, MT can inhibit the expression of IL-6 in hippocampus (Dong et al., 2016). IL-6 is a multifunctional pro-inflammatory cytokine that is produced in response to inflammatory stimuli and secreted by macrophages and monocytes, playing a key role in important biological pathways underlying stress and stress-induced depression (Anderson et al., 2013). Previous studies also have demonstrated that the serum levels of IL-6, interleukin-1β, TNF-α, and other pro-inflammatory cytokines were increased in depression patient under long-term chronic stress (Leonard and Maes, 2012). High level of IL-6 increases the risk of developing late-life depression (Setiawan et al., 2015) and is associated with treatment resistance of depression patients. Vogelzangs et al. (2014) further reported that measuring of plasma level of pro-inflammatory factor IL-6 can reflect the efficacy of antidepressant. Injection of IL-6 into rat amygdala and hippocampus could induce depression-like symptoms (Anderson et al., 2013). These results suggested that change of IL-6 level is important in development of depression and assessment of the efficacy of antidepressant.

Melatonin supplements also can reduce elevated plasma homocysteine (hcy) level. Hcy is a sulfurized amino acid derived from methionine and is crucial in mediating methylation and maintaining the biochemical balance within the central nervous system. A higher hcy level can enhance the oxidative stress, activation of *N*-methyl-D-aspartate (NMDA) glutamate receptor, and inhibition of sodium–potassium ATPase function, resulting in apoptosis of neurons to cause the death of hippocampal neurons in human brain (Lee et al., 2013). High serum hcy level is closely associated with high risk of depression, and lower hcy level can reduce the incidence of depression (Chellappa and Ramaraj, 2009). Hcy treatment could induce occurrence of depression (Ferlazzo et al., 2008). However, whether serum hcy level could be used to diagnose the occurrence and recurrence of depression remains unknown.

Circulating IL-6 can also promote production of acute phase protein such as C-reactive protein (CRP), which can trigger classical pathway of complement (Bilbo and Schwarz, 2012). Complement system plays an important role in innate immune system and inflammation, and alternation of complement components may contribute to the pathogenesis of depression by participating in inflammation (Wei et al., 2017). Complement C3 is the main activator in complement pathways and has a close correlation with brain development, plasticity, and brain dysfunction. Research data from human postmortem brain samples and animal studies have demonstrated an important role for C3 in mediating depressive behaviors (Lanfumey et al., 2013). More importantly, knockout of complement component 3a receptor in chronic stress-induced depression model mice attenuates chronic stress-induced monocyte infiltration and depressive-like behavior (Crider et al., 2018). Similarly, C4 was also found to be involved in the neural synapse elimination and to play a potential role in MDD either by participating in inflammation or regulating neural functions (Bialas and Stevens, 2013). However, whether the complement system could be used for the diagnosis of depression remains unclear. More guidance in the diagnosis and treatment of depression through monitoring the levels of complements is needed.

In this study, we detected five indicators (MT, IL-6, hcy, C3, and C4) in first-episode depression patients, recurrent depression patients, and the healthy group. The results showed that serum MT, IL-6, and hcy levels in the first-episode group and recurrent group were significantly higher than those in the control group, supporting that serum MT, IL-6, and hcy levels were correlated with pathogenesis of depression.

## Materials and Methods

### Subjects

A total of 95 patients with depression were recruited from the Department of Psychiatry of the Second Xiangya Hospital of Central South University between November 2018 and March 2019. The patients were aged 16–65 years old, including 37 males and 58 females (Table 1). Meanwhile, 45 healthy controls (HC) were recruited from the health management center of the Second Xiangya Hospital of Central South University. As shown in the Table 1, the HC were 23–62 years old, including 17 males and 28 females, and had no mental illness and other family history of hereditary psychosis. The patients were divided into two subgroups: first-episode group (43 cases) and recurrent group (52 cases). These patients are in compliance with the fifth edition of Diagnostic Statistics of Neuropsychiatrics published by the American Psychiatric Association. The inclusion criteria for patients were as follows: (1) the scores from 24 Hamilton Depression Scale (HAMD) greater than or equal to 20 points; (2) no other mental illnesses and neurological diseases, and there are no obvious abnormalities in examination results of three routine, biochemical, immune, and physical; (3) no history of alcohol abuse; and (4) no immunosuppressants or immunopotentiators used in the past 6 months. The exclusion criteria for patients were as follows: (1) severe physical illness within other tissue and organ; (2) unknown drug usage; (3) five or more drugs were taken in the short term; and (4) pregnant or lactating women. Our study was supported by the Ethics Committee of the Second Xiangya Hospital of Central South University. Informed consent was signed by all patients or their legal guardians and HC.

**TABLE 1:** Demographic data for depression patients and control group.

	First-episode depression patients	Recurrent depression patients	Control group	*t*/χ^2^	*P* value
Sex	*N* (%)	*N* (%)	*N* (%)	0.032	0.984
Male	15(34.9%)	22(42.3%)	17(37.8%)		
Female	28(65.1%)	30(57.7%)	28(62.2%)		
Total	43	52	45		
Age (mean ± SE)	34.13 ± 2.86	41.73 ± 2.53	40.41 ± 1.71	2.439	0.091

### Sample Collection and Processing

Peripheral venous bloods of all patients and HC were collected from each participant at 08:00 a.m. after overnight fasting. Serum samples were stored at −80°C until analysis after centrifuging at 3000 rpm for 10 min. All samples were thawed to room temperature before testing. Serum MT levels were measured using enzyme-linked immunosorbent assay (ELISA) kits (ArigoBiolaboratories, Taiwan, China). Serum IL-6 levels were measured with electrochemical luminescence in Cobas E602 (Roche, Germany). Serum hcy levels were measured with Hitachi 7600 automatic biochemical analyzer. Serum complements C3 and C4 levels were measured with immuno-scatter turbidimetric assay in automatic specific protein analyzer (Beckman Court).

### Statistical Analysis

SPSS v21.0 (IBM) statistical software was used to perform statistical analysis. All values are presented as mean ± standard deviation. Two-group comparisons of the continuous variables were performed using unpaired two-tailed Student’s *t* test. One-way analysis of variance (ANOVA) was used to perform multiple-group comparisons. *P* value < 0.05 was considered statistically significant.

## Results

Table 1 summarizes the demographic and clinical characteristics of the cases on enrollment. The mean age of the first-episode depression patients was 34.13 ± 2.86 years old, and 34.9% of patients were males. The mean age of the recurrent depression patients was 41.73 ± 2.53 years old, and 42.3% of patients were males. Similarly, the mean age of the control group was 40.41 ± 1.71 years old, and 37.8% were males. There was no significant difference in terms of age and gender between the three groups (*t* = 2.439, *P* = 0.091; χ^2^ = 0.032, and *P* = 0.984).

### Changes of Serum MT Levels in the First-Episode, Recurrent, and Control Group

**FIGURE 1:**
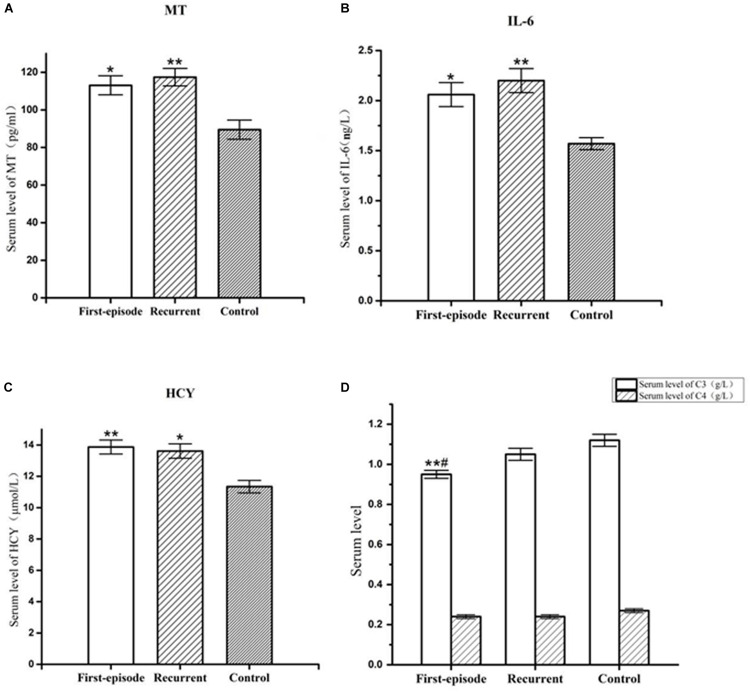
Representative of the serum MT,IL-6, hcy, C3, and C4 levels in first-episode group, recurrent group, and control group. **(A)** Changes of serum MT concentration of patients with first-episode depression (*P < 0.05) and recurrent depression (**P < 0.01) in comparison to that of control group. **(B)** Changes of serum IL-6 concentration of patients with first-episode depression (*P < 0.05) and recurrent depression (**P < 0.01) in comparison to that of control group. **(C)** Changes of serum hcy concentration of patients with first-episode depression (**P < 0.01) and recurrent depression (*P < 0.05) in comparison to that of control group. **(D)** Changes of serum C3 concentration of patients with first-episode depression in comparison to recurrent depression (^#^*P* < 0.05) and control group (**P < 0.01). There was no significant difference in serum C4 concentration between each group.

The serum MT levels in the first-episode group and recurrent group (113.08 ± 5.06 pg/ml, 117.43 ± 4.63 pg/ml) were significantly higher (*P* = 0.027; *P* = 0.009) than those in the control group (89.50 ± 5.10 pg/ml). However, there was no significant difference in serum MT levels between the first-episode group and recurrent group (*P* = 0.644; Figure 1).

### Changes of Serum IL-6 Levels in the First-Episode, Recurrent, and Control Group

Serum IL-6 levels in the first-episode group and recurrent group (2.06 ± 0.12 ng/L, 2.20 ± 0.12 ng/L) were significantly higher (*P* < 0.038; *P* = 0.008) than those in the control group (1.57 ± 0.06 ng/L). However, there was no significant difference in serum IL-6 levels between the first-episode group and the recurrent group (*P* = 0.508; Figure 1).

### Changes of Serum Hcy Levels in the First-Episode, Recurrent, and Control Group

Serum hcy levels in the first-episode group and the recurrent group (13.87 ± 0.45 μmol/L, 13.61 ± 0.46 μmol/L) were significantly higher (*P* = 0.009; *P* = 0.019) than those in the control group (11.34 ± 0.40 μmol/L). However, there was no significant difference in serum hcy levels between the first-episode group and the recurrent group (*P* = 0.760; Figure 1).

### Changes of Serum Complement Factors C3 and C4 Levels in the First-Episode, Recurrent, and Control Group

Serum complement factor C3 levels in the first-episode group (0.95 ± 0.03 ng/L) was also significantly lower than those the in recurrent group (1.05 ± 0.03 ng/L) and control group (1.12 ± 0.03 ng/L; *P* = 0.044; and *P* = 0.003). However, there was no significant difference in serum complement factor C3 level between the recurrent group and the control group (*P* = 0.215; Figure 1). There was no significant difference in serum complement factor C4 level between the first-episode group, recurrent group, and control group (0.24 ± 0.01 ng/L, 0.24 ± 0.01 ng/L, and 0.27 ± 0.01 ng/L; *F* = 1.526, *P* = 0.223; Figure 1).

## Discussion

In this study, we found that the serum levels of MT, IL-6, and hcy in the first-episode group and recurrent group were significantly higher than those in control group, and serum levels of C3 in the first-episode group were significantly lower than those in the recurrent group and control group. The metabolism of MT in peripheral blood could partially reflect depression disorder in the brain (Dmitrzak-Weglarz and Reszka, 2017). Rubin et al. (1992) have found that the concentration of nocturnal MT is significantly elevated in endogenous major depression patients when compared with that of the HC group. Srinivasan et al. also have reported that MT levels were increased in MDD patients. Here, our experiment results showed significantly higher serum MT levels in depression patients in comparison to those in the control group, which is consistent with previous reports. The higher serum MT levels in first-episode depression patients might be attributed to the pharmacological action of antidepressants (Srinivasan et al., 2012). However, Bumb et al. (2016) also found significantly reduced serum MT levels in major depressive patients. The different results may be related to the different time of serum collection or distinct method for measuring of serum MT levels (Bumb et al., 2016). In particular, the serum levels of MT in the recurrent group were significantly higher than those in the control group. Previous studies reported that antidepressant drugs (fluvoxamine and imipramine) can cause significant elevation of urinary MT metabolites in treatment responders among major depression patients (Miller et al., 2001). In this study, the patients were treated with lexapro, seroquel, and sertraline. Therefore, we speculate that the increased serum MT levels in the recurrent depression patients may also be related to the pharmacological action of antidepressant drugs.

Interleukin-6 is a multifunctional pro-inflammatory cytokine produced by monocytes in periphery and secreted by microglia in the central nervous system (Rothaug et al., 2016) and can cross the blood–brain barrier-deficient areas. Eyre et al. (2016) found significantly higher concentrations of IL-6 in the blood of depressed subjects in comparison with HC. Dunjic-Kostic et al. (2013) also reported that serum IL-6 was significantly elevated in melancholic depressive patients compared to HC. Liu et al. (2012) reported that blood levels of IL-6 were significantly higher in MDD patients than those in HC. In this study, we found that serum IL-6 levels in depressive patients are significantly higher than those in the control group, which is consistent with the results reported by previous studies (Maes et al., 1993; Liu et al., 2012; Vogelzangs et al., 2016). Increased IL-6 activity in severe depression is related to hyperactivity of the HPA axis (Maes et al., 1993). Therefore, we speculate that the increased serum IL-6 levels in first-episode depression patients may be attributed to excessive activation of the HPA axis. Plasma pro-inflammatory factor IL-6 of depression patient who was treated with depressant drugs is higher than that in the control group (Vogelzangs et al., 2016). Therefore, higher serum IL-6 level in the recurrent group compared with that in control group may be due to the use of traditional antidepressant drugs.

Hyperhomocysteinemia can disturb normal neurological function in many ways like DNA damage, oxidative stress, and inflammation, inducing occurrence of depression. Bottiglieri et al. (2000) reported that 52% of severe DSM III depression patients have higher total plasma hcy than the HC group. Patients who were suffering from recurrent MDD have higher hcy levels compared to those in remission (Lok et al., 2014). Elevated serum hcy concentrations are associated with lifetime MDD and particularly with remitted MDD among men (Nabi et al., 2013). Our study found significantly higher serum hcy levels in patients with depression in comparison to control group, which is consistent with previous studies (Bottiglieri et al., 2000; Nabi et al., 2013; Lok et al., 2014). Folic acid and vitamin B12 were confirmed to be administered in depression treatment by decreasing the hcy level. Therefore, the high hcy level in first-episode and recurrent depression patients might be attributed to the deficiency of vitamin B12 and folic acid.

Complement C3 is the pivotal factor in connecting between classical and alternative pathway of complement system and is the most abundant complement factors in serum. Nguyen et al. (2016) reported lower complement expression in the periphery in conjunction with depressive symptoms post-stroke. Here, our results showed that serum C3 levels in the first-episode depression group are lower than those of the control group, which is consistent with the result reported by Nguyen et al. For the decreased serum C3 levels, we speculate that the reason might be that dysfunction of humoral immune, such as inflammation-induced enhancement of B cell function in patients with first-episode depression, led to increased clearance of immune complexes *in vivo*. However, change of C4 levels in our study is not obvious between first-episode depression patients and the control group. We can speculate that the three complement pathways cannot maintain balance in depression patients but the specific disorder mechanism is not detailed (Strenn et al., 2015). Besides, it is worth mentioning that the serum C3 level in the first-episode group is significantly lower than that in the recurrent group, which is presumed to be related to the relief of depressive symptoms after a period of anti-depressive drug treatment because C3 was significantly associated with response to antidepressants (Chan et al., 2016).

Besides, our results also demonstrated that there were no significant differences between first-episodic group and recurrent group in serum MT, IL-6, hcy, and C4 levels. The reasons may be complicated. Therefore, more studies are needed to clarify the underlying mechanism.

## Conclusion

In summary, our results suggested that MT, IL-6, hcy, and complement factor C3 may be of great importance in pathogenesis process of depression. On the basis of the comparison of the change of serum MT levels in our study with that reported in previous research, we can infer that the disease stage may affect serum MT levels in depressed patients to a large extent. Furthermore, the changes of serum C3 levels between first-episode and recurrent patients may imply that more researches are needed to be done in order to deeply understand the detailed mechanism of complement disorder in patients with depression.

## Data Availability Statement

The datasets generated for this study are available on request to the corresponding author.

## Ethics Statement

The studies involving human participants were reviewed and approved by the ethics committee of the Second Xiangya Hospital of Central South University. Written informed consent to participate in this study was provided by the participants’ legal guardian/next of kin.

## Author Contributions

HT and YL were responsible for the design of the experiment, conducting the statistical analysis of the resulting data, and writing, revising, editing, and submitting the manuscript. XC and JF collected the clinical samples and data and conducted the statistical analysis of the resulting data. HZ and QY collected the literature and wrote the draft. All authors contributed to the article and approved the submitted version.

## Conflict of Interest

The authors declare that the research was conducted in the absence of any commercial or financial relationships that could be construed as a potential conflict of interest.
